# Effect of geographic accessibility to primary care on treatment status of hypertension

**DOI:** 10.1371/journal.pone.0213098

**Published:** 2019-03-04

**Authors:** Kenta Okuyama, Kenju Akai, Tsunetaka Kijima, Takafumi Abe, Minoru Isomura, Toru Nabika

**Affiliations:** 1 Center for Community-based Healthcare Research and Education (CoHRE), Organization for Research and Academic Information, Shimane University, Izumo City, Shimane, Japan; 2 Department of General Medicine, Faculty of Medicine, Shimane University, Izumo City, Shimane, Japan; 3 Faculty of Human Sciences, Shimane University, Matsue City, Shimane, Japan; 4 Department of Functional Pathology, Faculty of Medicine, Shimane University, Izumo City, Shimane, Japan; Universidad Miguel Hernandez de Elche, SPAIN

## Abstract

Although primary care access is known to be an important factor when seeking care, its effect on individual health risk has not been evaluated by an appropriate spatial measure. This study examined whether geographic accessibility to primary care assessed by a sophisticated form of spatial measure is associated with a risk of hypertension and its treatment status among Japanese people in rural areas, where primary care is not yet established as specialization. We used an enhanced two-step floating catchment area method to calculate the neighborhood residential unit-level primary and secondary care accessibility for 52,029 subjects who participated in the 2015 annual health checkup held at 15 cities in Shimane Prefecture. Their hypertension level and treatment status were examined cross-sectionally with their neighborhood primary care and secondary care accessibility (computed with two separate distance-decay weight: slow and quick) by multivariable logistic regression controlling for demographics and neighborhood income level. The findings showed that greater geographic accessibility to primary care was associated with a decreased risk of hypertension in both slow and quick distance-decay weight, odds ratio (OR) = 0.989 (95% Confidence Interval (CI) = 0.984, 0.994), OR = 0.989 (95%CI = 0.984, 0.993), respectively. On the other hand, better secondary care accessibility was associated with an increased risk of hypertension and untreated hypertension; however, the effect of secondary care was mitigated by the effect of primary care accessibility in both slow and quick distance-decay model, hypertension: OR = 0.974 (95% CI = 0.957, 0.991), OR = 0.981 (95%CI = 0.970, 0.991), untreated hypertension: OR = 0.970 (95%CI = 0.944, 0.996), OR = 0.975 (95%CI = 0.959, 0.991), respectively.

In addition, the results revealed that young and fit people were at a higher risk of untreated hypertension, which is a unique finding in the context of the Japanese healthcare system. Our findings indicate the importance of primary care even in Japan, where it is not yet established, and also emphasize the need for a culturally specific perspective in health equity.

## Introduction

Access to care is an important factor that determines the health of the overall population [1]. Easy access to care is known to lower mortality and morbidity [2, 3], and primary care services function to reduce burdens on patients in terms of time and cost while offering appropriate treatments, thereby improving the health of the population [4]. Despite evidence that emphasizes the importance of primary care for population health, primary care is not yet well-established in Japan. The Organization for Economic Co-operation and Development (OECD) evaluated the quality of the Japanese healthcare system in 2015. Though the OECD’s evaluation noted the reputedly-long life expectancy of the Japanese population, it also pointed out that compared to other OECD countries, Japanese people experience considerably longer hospital stays and higher healthcare expenditures. The report indicated that the lack of a distinct primary care specialty is one reason, and it is critical for a rapidly aging population in which a preventive and holistic approach to healthcare is needed [5]. Primary care in Japan is provided by semi-generalists/semi-specialists who have been working as generalists in the community with unspecified amount of time to train as generalists after they left hospital practice. On the other hand, generalists in Europe are required to complete specialized training in family medicine, and primary care is recognized as a distinct medical specialty. Access to care, which is an important principle of primary care, consists of multiple domains: approachability, acceptability, availability and accommodation, affordability, and appropriateness [1, 6]. Availability, which entails geographic accessibility, is crucial, especially in rural settings. However, healthcare resources, including both facilities and physicians, are unequally distributed across regions; this remains a significant issue globally [7, 8]. In Japan, though unequal access to healthcare resources and regional health disparity are among the major public health problems, studies on geographic accessibility to healthcare are fairly limited [9].

Hypertension is one of the most prevalent chronic conditions worldwide as well as in Japan. According to the Patient Survey by the Ministry of Health, Labour and Welfare Japan, as of 2014, more than 10 million people in Japan experience hypertension [10]. Although hypertension does not have immediate effects on health, it can lead to fatal chronic diseases such as cardiovascular disease and stroke [11]. Blood pressure should be controlled through anti-hypertensive medication and lifestyle modification to avoid such disease complications [12]. Anti-hypertensive medication alone can bring about a nearly 50% relative risk reduction in the incidence of heart failure, a 30%–40% relative risk reduction in the incidence of strokes, and 20%–25% relative risk reduction in the incidence of myocardial infractions [12]. Geographic proximity to healthcare is one of the essential factors for determining whether or not people seek care [1]. The European Society for Quality and Safety in Family Practice (EQuiP), which is part of the European network of the World Organizations of Family Doctors, also emphasizes the importance of geographic accessibility to medical facilities and specialists for achieving equity in health [13]. Meanwhile, numerous studies have proposed methodologies for ensuring geographic healthcare accessibility and have identified a regional maldistribution of healthcare resources [14, 15]. The most common method of spatial measure is the two-step floating catchment area method (2FCA), and it accounts for both physical distance and supply-demand ratio by using Geographic Information System (GIS). This method is considered to be a better accessibility measure than traditional approaches of either supply-demand ratio within an administrative boundary or closest distance to a specific facility. In a recent study on access to care and control of hypertension, poor access to care was associated with an increased risk of uncontrolled hypertension [16]. However, limited studies have used this spatial measure to examine the association between geographic accessibility to healthcare and individual risk of disease, and none for hypertension [17].

We hypothesized that although primary care is not well-established in Japan, its geographic accessibility factors into hypertension risk and is a determinant for whether or not people seek care, especially for those who reside in rural settings. The aims of this study were to examine whether geographic accessibility to primary care facilities is important for lowering the risk of hypertension and untreated hypertension status, and to provide useful information to guide ongoing primary care policy.

## Methods

### Data

This study used cross-sectional data of employees with employer-based insurance living in 15 municipalities of the Shimane Prefecture, who participated in the annual health checkup at their workplace in 2015. The data were provided by The Shimane Environment & Health Public Corporation, which is the governmental institution that conducts annual health checkups in Shimane Prefecture. The Shimane Prefecture is 6708.24 km^2^, and in 2015, its population was 694,352 (male: 333,112, female: 361,240). The data of this study (n = 57,858) cover approximately 10% of the population over 15 years old: 608,296. The subjects’ individual addresses were available at the centroid point of administrative unit, “Chou Aza,” which refers to the smallest regional unit in the Japanese census and is equivalent to the census boundary of other countries. We geocoded subjects over 30 years old and assigned a unit-level healthcare accessibility score to each individual. The study protocol and procedure were approved by the Ethics Committee at Shimane University School of Medicine in 2015.

### Spatial measure for healthcare accessibility

We used the enhanced two-step floating catchment area method (E2FCA) to assess geographic accessibility to primary and secondary care facilities in each Chou Aza unit. Both primary and secondary care facilities were exclusively those with any internal medicine department. Facilities with only specific medicine such as ophthalmology, obstetrics and gynecology, dermatology were not included. Primary care facility is commonly named in clinic, and formally defined as a clinic with less than 20 beds in Japan. Secondary care facility is defined as a hospital with 20 and more beds. Primary care facilities are used by people who have many types of health issues except for acute emergencies or those requiring operations. Secondary care facilities are used by people who have health issues as well as severe conditions such as detailed investigations of the ischemic heart disease, and abdominal operations can be conducted. The E2FCA is a spatial measure for healthcare that was originally developed in 2003 [18] and was later enhanced [14]. Though there is no optimal index established, the E2FCA is known as a sophisticated form of spatial measure for considering both the supply-demand ratio and distance as well as distance-decay weighting function, as it appropriately assesses the interaction of the provider with the population based on distance-decay theory. Briefly, the E2FCA score is calculated as follows.

$$R_{j}=\frac{1}{\sum_{k\in \{d_{kj}\in D_{r}\}}P_{k}W_{r}}$$

Step 1: From each healthcare facility *j*, a multiple network buffer *D*_*r*_ was created. The number of people *P* within neighborhood *k* covered by each buffer *D* was summed. *W* is the weight based on distance-decay function, and it was applied to the summed population in each buffer *r*. As our supply was each healthcare facility, 1 was used as a numerator. *R* is the provider-to-population ratio for each healthcare facility *j*.

$$A_{i}^{F}=\sum_{j\in \{d_{ij}\in D_{r}\}}R_{j}W_{r}$$

Step 2: From the centroid point of the residential administrative unit, multiple network buffers *D*_*r*_ were created. The provider-to-population ratios *R* calculated in step 1 for each healthcare facility *j* within each buffer *D* were summed. The same distance-decay weight *W* was applied to the summed provider-to-population ratios in each buffer *r*. *A* is the final E2FCA score for each administrative unit *i*.

**Fig 1:**
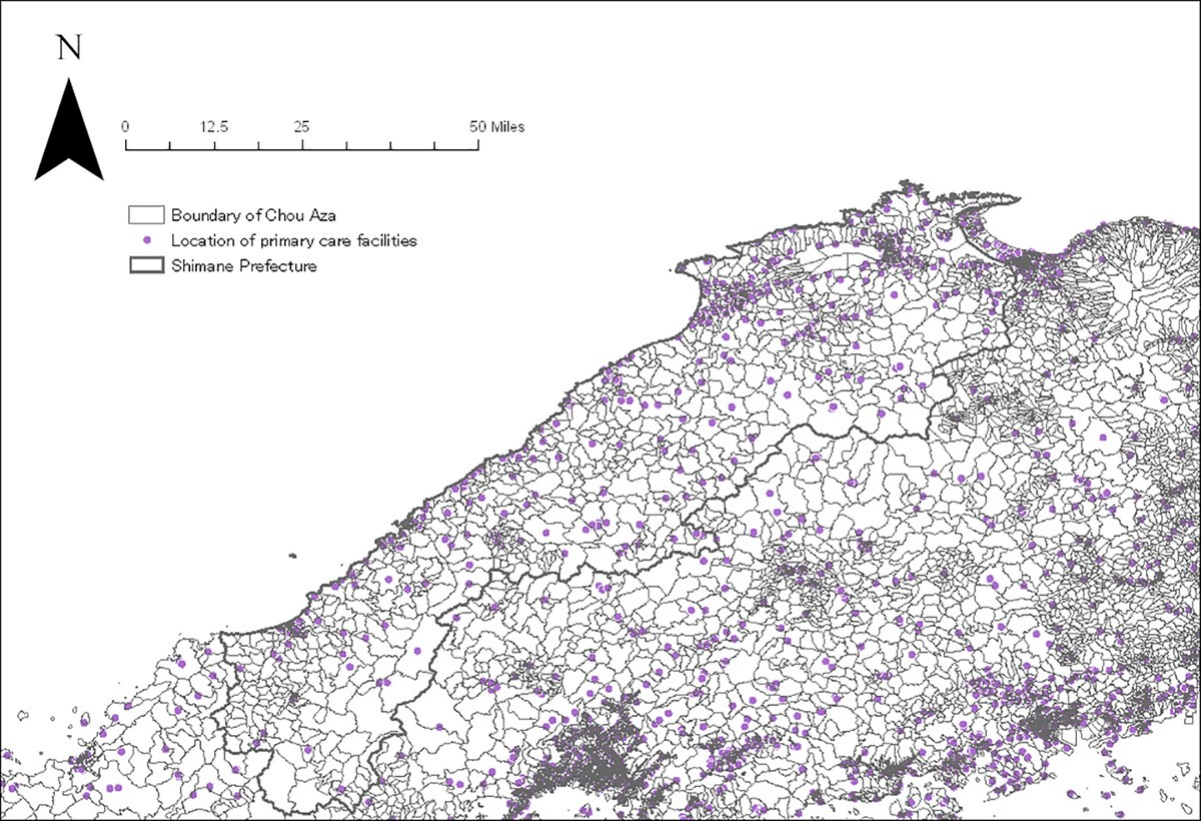
Location of primary care facilities in Shimane and four peripheral prefectures.

**Fig 2:**
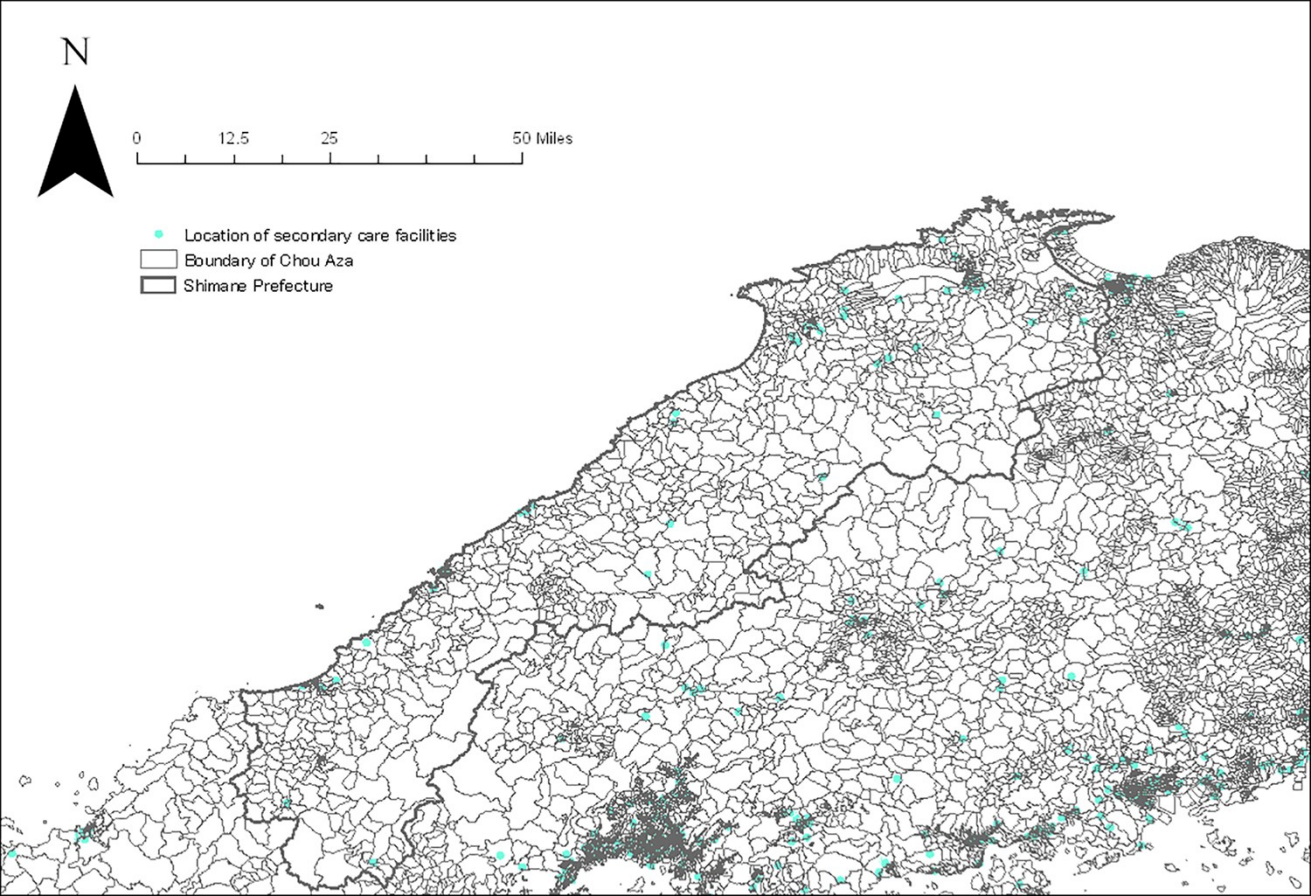
Location of secondary care facilities in Shimane and four peripheral prefectures.

**Fig 3:**
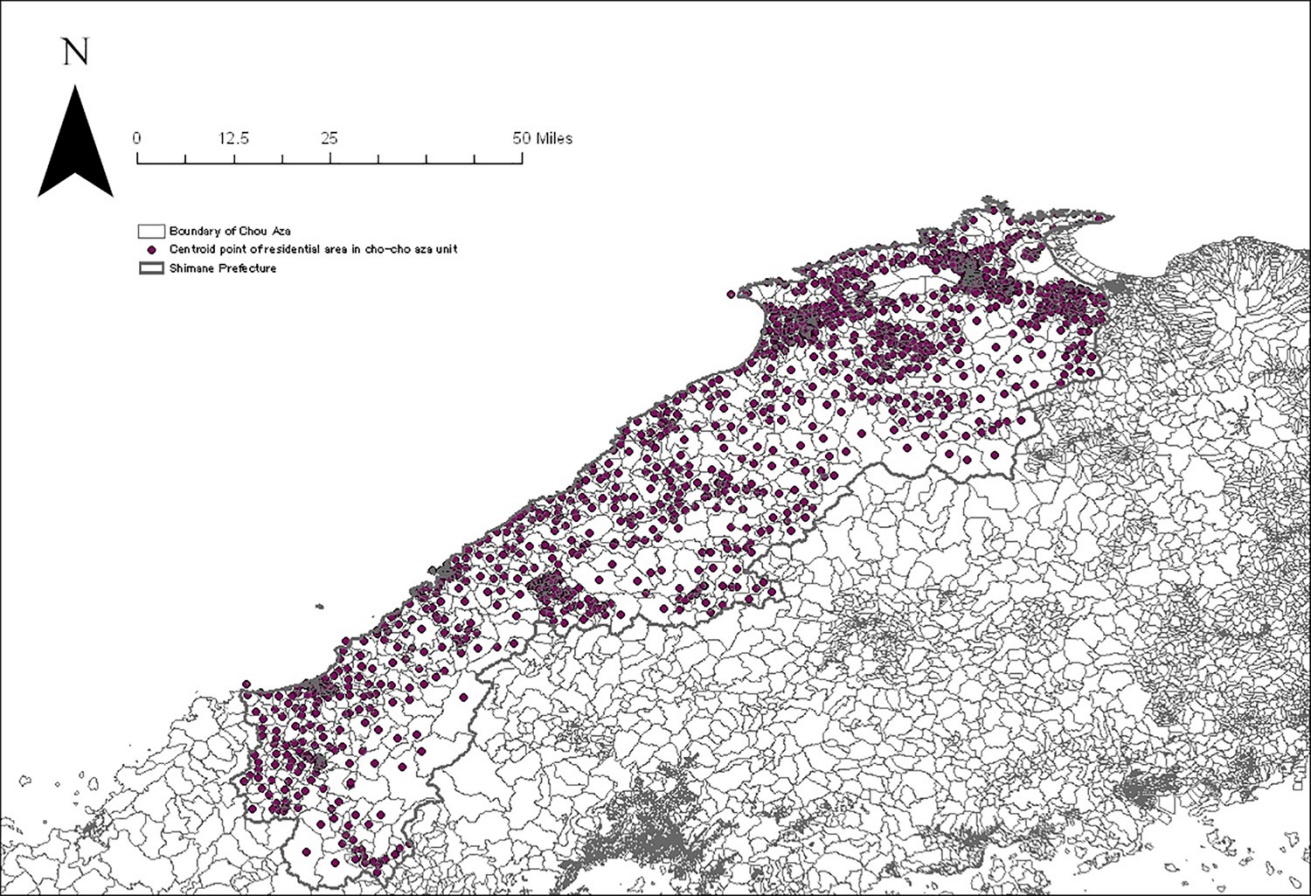
Centroid point of the residential area in each administrative unit.

We created four catchment zones with a minimum of 10 minutes up to a maximum of 60 minutes with discrete distance-decay function following a prior study in rural settings [15]. The following distance-decay weights *W* for each buffer zone *r* were applied for each catchment zone: slow distance-decay—*W*_1_ = 1, *W*_2_ = 0.80, *W*_3_ = 0.55, and *W*_4_ = 0.15; and quick distance-decay—*W*_1_ = 1, *W*_2_ = 0.60, *W*_3_ = 0.25, and *W*_4_ = 0.05. As there is no consensus about which distance-decay weight is superior to the other, we applied both weights and computed accessibility scores separately. Thus, E2FCA scores for each administrative unit were computed for primary (n = 4,870) and secondary care facilities (n = 588) with internal medicine specializations that are located in Shimane as well as four peripheral prefectures (Figs 1 and 2). The E2FCA scores for primary and secondary care facilities were assigned for geocoded samples. There were a few units with extreme values for the E2FCA score for primary care, and those areas were assigned 99th percentile value as in a prior study [19]. Data of healthcare facilities as of 2014 were taken from the National Land Numerical Information (NLNI), which is publicly available GIS data administered by the National Land Information Division, National Spatial Planning, and Regional Policy Bureau of Japan. Road network data from the Esri Japan Corporation in Tokyo, Japan as of 2014 were used in the network analysis. Administrative unit-level population data based on the 2015 Population Census of Japan was extracted from ArcGIS data collection standard pack 2018 by Esri Japan. Given that 77.5% of land in Shimane, our study area, was covered by forest, instead of using the centroid point of administrative unit, we used the centroid point of residential area for each administrative unit by excluding forests, rivers, and lakes from land use data of NLNI 2015 (Fig 3). Arc GIS Pro version 2.0 (Esri Inc., Redland, CA, USA) was used for the spatial measure analysis.

### Outcome

We set two separate outcome variables. First, hypertension status was defined based on the following criteria: systolic blood pressure greater than 140, diastolic blood pressure greater than 90, or taking anti-hypertensive medication. Second, among those who had hypertension, untreated hypertension status was defined based on the following criteria: systolic blood pressure greater than 140 or diastolic blood pressure greater than 90, and not taking anti-hypertensive medication.

### Covariates

#### Demographics

The data included the following demographic information: gender, age, current smoking status (yes/no), current drinking status (yes/no), and body mass index (BMI). The information was collected via face-to-face interviews during the health checkup, and BMI was calculated based on height and weight objectively measured by healthcare practitioners.

#### Neighborhood income

We used the mean income of each administrative unit as the estimate of socioeconomic status. Data were created using KOKUSAI KOGYO CO. LTD based on the Census, Housing and Land Survey and the National Survey of Family Income and Expenditure as of 2010.

### Statistical analysis

We applied a multivariable logistic regression model to predict the odds of hypertension (vs. no-hypertension) and untreated hypertension (vs. treated) by E2FCA scores for primary and secondary care solely (Model 1) and included them as an interaction term (Model 2). We adjusted demographic characteristics of subjects including age, gender, smoking status, drinking status, BMI, and neighborhood mean income. We excluded the subjects with missing data after diagnosing the patterns of missing information, and the proportion of missing values to total subjects was considerably low (0.012%). All statistical analysis was conducted with R version 3.4.4.

## Results

**Fig 4:**
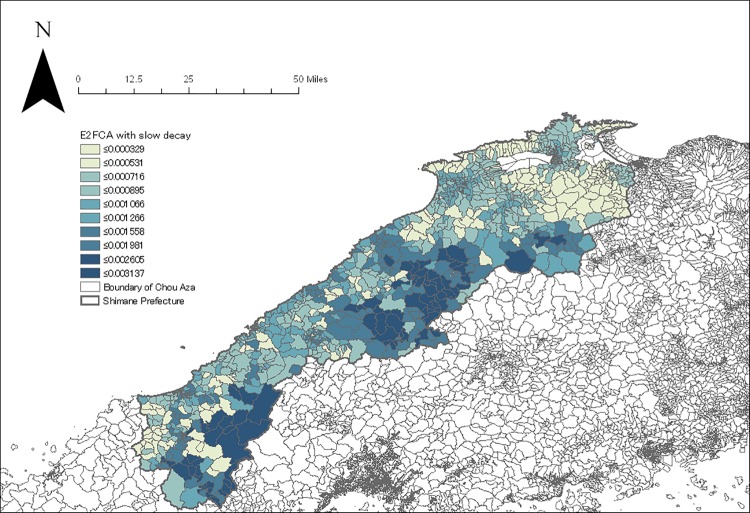
The E2FCA score for primary care facilities with slow distance-decay.

**Fig 5:**
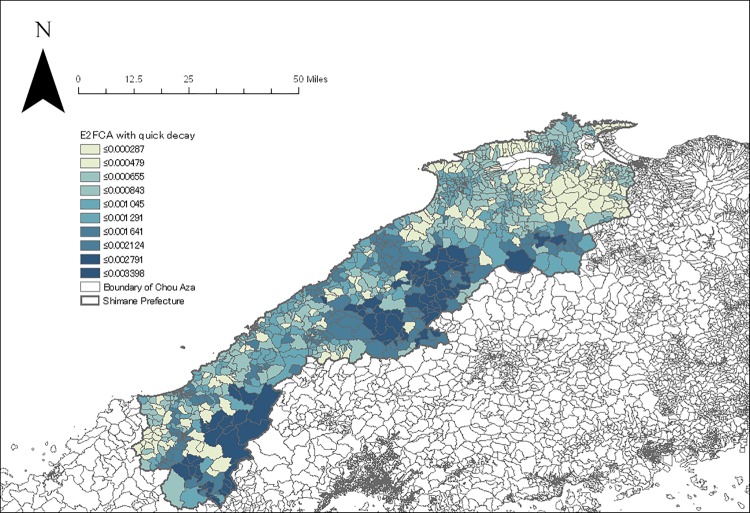
The E2FCA score for primary care facilities with quick distance-decay.

**Fig 6:**
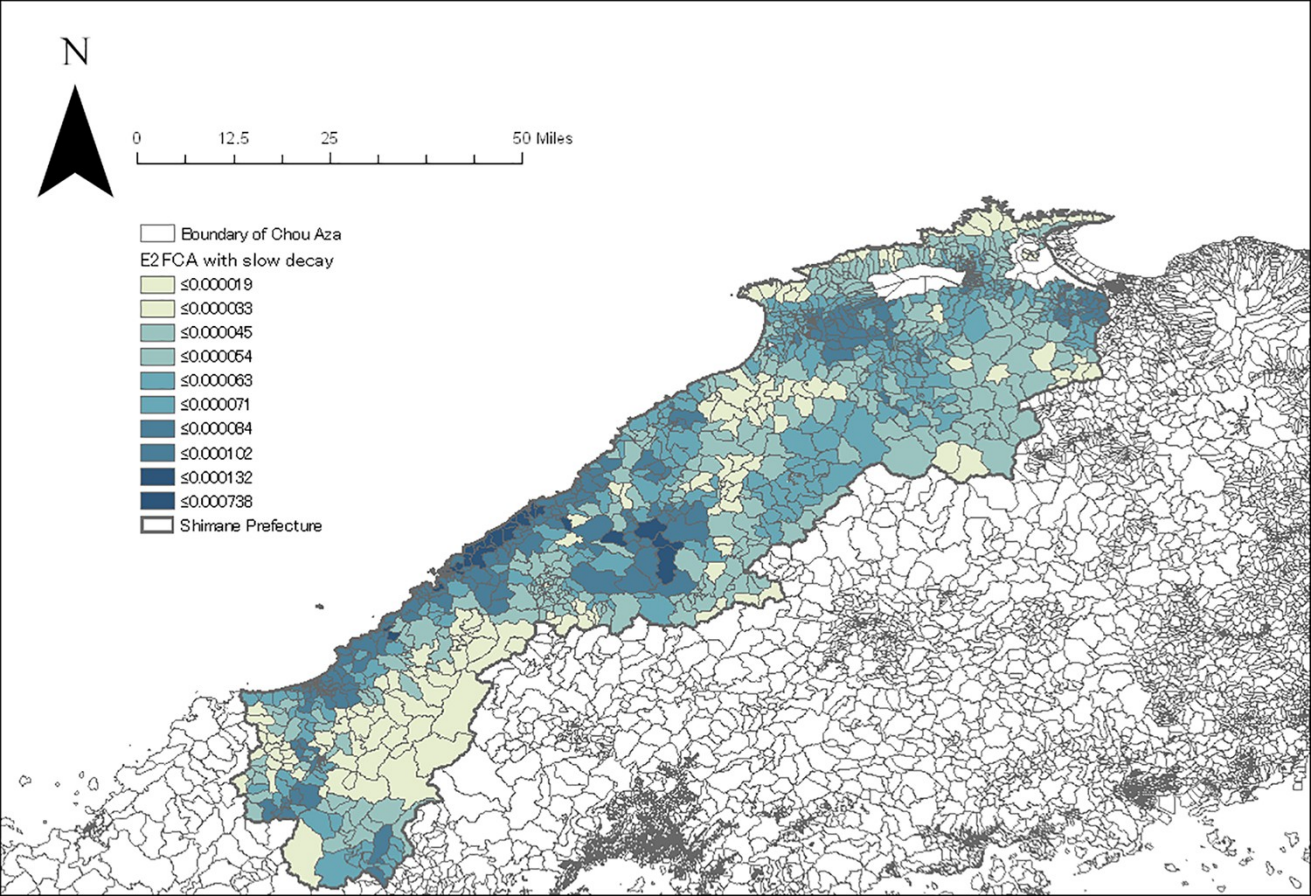
The E2FCA score for secondary care facilities with slow distance-decay.

**Fig 7:**
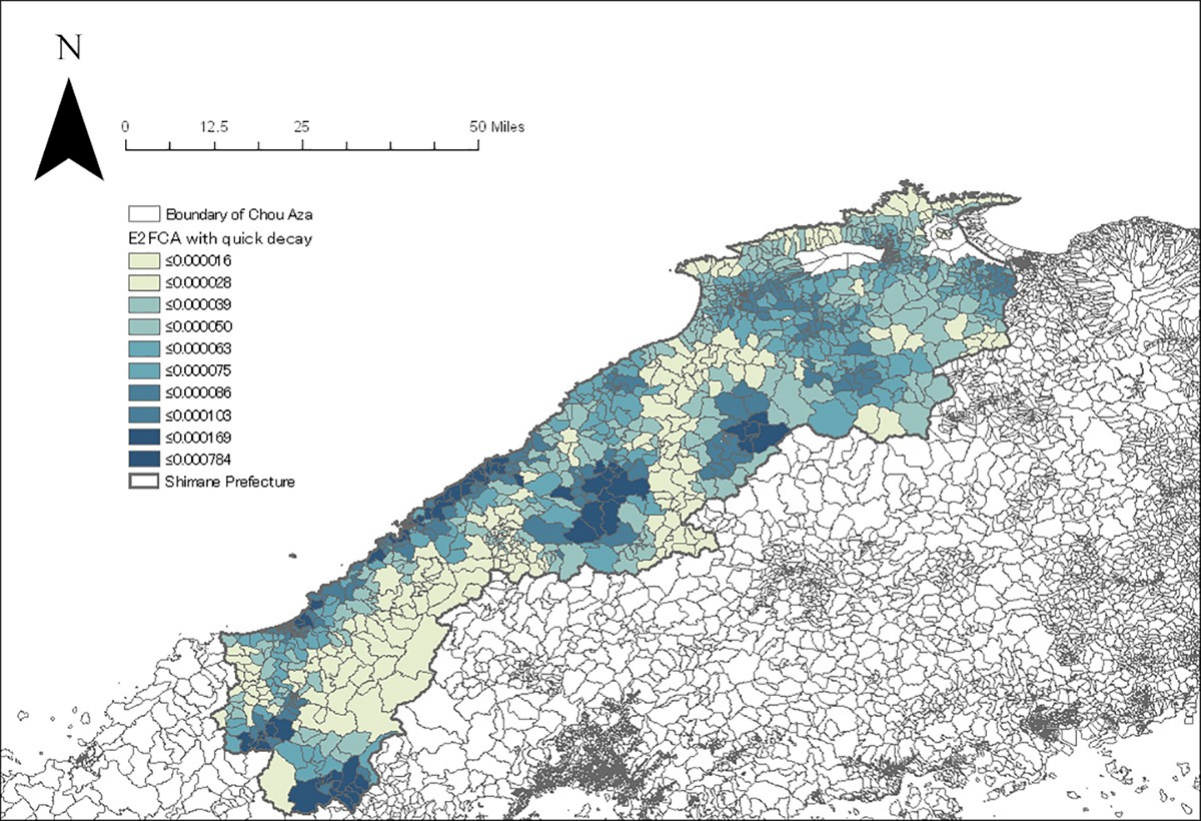
The E2FCA score for secondary care facilities with quick distance-decay.

The geocoding process resulted in locating 52,029 subjects in a total of 1210 Chou Aza units. The E2FCA scores for primary and secondary care facilities with slow and quick distance-decay were assigned for each sample according to their residential units (Figs 4–7). Tables 1 and 2 show the basic characteristics of study samples by hypertension and untreated hypertension statuses. There were 14,369 (27.6%) subjects who had hypertension (Table 1). There were significantly more males (vs. females), smokers (vs. non-smokers), and drinkers (vs. non-drinkers) among those with hypertension than those without hypertension. Age and BMI were higher among those with hypertension than those without hypertension. Neighborhood mean income was significantly lower among those with hypertension than those without hypertension. Accessibility (E2FCA score) to primary and secondary care were significantly higher among those with hypertension than those without hypertension for both slow and quick distance-decay. Among people with hypertension, 6693 (46.6%) subjects had untreated hypertension (Table 2). There were significantly more males (vs. females), smokers (vs. non-smokers), and drinkers (vs. non-drinkers) among those with untreated hypertension than those with treated hypertension. Age and BMI were lower among those with untreated hypertension than those with treated hypertension. Neighborhood mean income was significantly lower among those with untreated hypertension than those with treated hypertension. E2FCA scores for primary care were significantly lower among those with untreated hypertension than those with treated hypertension for both slow and quick distance-decay. On the contrary, E2FCA scores for secondary care were higher among those with untreated hypertension than those with treated hypertension.

**Table 1:** Descriptive statistics of study samples by hypertension status.

	Hypertension status		
	hypertension	no-hypertension	p
N	14369	37660	
Gender = Male/Female (%)	9390/4979 (65.3/34.7)	19162/18498 (50.9/49.1)	<0.001
Age (years) (mean (sd))	58.10 (10.74)	47.97 (10.94)	<0.001
BMI (mean (sd))	24.16 (3.89)	22.26 (3.36)	<0.001
Smoking = No/Yes (%)	7120/7249 (49.6/50.4)	21627/16033 (57.4/42.6)	<0.001
Drinking = No/Yes (%)	6046/8323 (42.1/57.9)	20484/17176 (54.4/45.6)	<0.001
Mean neighborhood income (10,000 yen) (mean (sd))	459.07 (54.85)	464.82 (54.86)	<0.001
E2FCA score for Primary care with slow distance-decay *10^−4^ (mean (sd))	11.02 (10.48)	10.37 (7.23)	<0.001
E2FCA score for Secondary care with slow distance-decay *10^−4^ (mean (sd))	0.69 (0.24)	0.68 (0.22)	0.025
E2FCA score for Primary care with quick distance-decay *10^−4^ (mean (sd))	11.24 (11.11)	10.55 (7.72)	<0.001
E2FCA score for Secondary care with quick distance-decay *10^−4^ (mean (sd))	0.70 (0.33)	0.69 (0.30)	0.199

**Table 2:** Descriptive statistics of study samples by untreated hypertension status.

	Untreated hypertension status		
	untreated	treated	p
N	6638	7731	
Gender = Male/Female (%)	4513/2125 (68.0/32.0)	4877/2854 (63.1/36.9)	<0.001
Age (years) (mean (sd))	54.41 (10.51)	61.28 (9.89)	<0.001
BMI (mean (sd))	23.93 (3.91)	24.35 (3.85)	<0.001
Smoking = No/Yes (%)	3111/3527 (46.9/53.1)	4009/3722 (51.9/48.1)	<0.001
Drinking = No/Yes (%)	2672/3966 (40.3/59.7)	3374/4357 (43.6/56.4)	<0.001
Mean neighborhood income (10,000 yen) (mean (sd))	458.02 (55.41)	459.96 (54.36)	0.034
E2FCA score for Primary care with slow distance-decay *10^−4^ (mean (sd))	10.58 (8.56)	11.40 (11.86)	<0.001
E2FCA score for Secondary care with slow distance-decay *10^−4^ (mean (sd))	0.71 (0.24)	0.67 (0.24)	<0.001
E2FCA score for Primary care with quick distance-decay *10^−4^ (mean (sd))	10.76 (9.06)	11.65 (12.58)	<0.001
E2FCA score for Secondary care with quick distance-decay *10^−4^ (mean (sd))	0.72 (0.32)	0.68 (0.33)	<0.001

Table 3 shows the multivariable logistic regression result for dependent variables: hypertension and untreated hypertension status. The result without interaction term (Model 1) for hypertension status shows that the accessibility to primary care was negatively associated with the odds of having hypertension for both slow and quick distance-decay weight: odds ratio (OR) = 0.989 (95% Confidence Interval (CI) = 0.984, 0.994), OR = 0.989 (95%CI = 0.984, 0.993). The accessibility to secondary care was positively associated with the odds of having hypertension for both slow and quick distance-decay weight: OR = 1.281 (95% CI = 1.148, 1.430), OR = 1.758 (95%CI = 1.392, 2.222). The result of the advanced model with the interaction term of accessibility to primary and secondary care facilities (Model 2) for hypertension status shows that there was a negative association with the odds of having hypertension for both slow and quick distance-decay weight: OR = 0.974 (95% CI = 0.957, 0.991), OR = 0.981 (95%CI = 0.970, 0.991). The results for untreated hypertension status showed that there was not a significant association between the accessibility of primary care and the odds of having untreated hypertension for the models without interactions in both slow and quick distance-decay weight, OR = 0.999 (95%CI = 0.992, 1.007), OR = 0.993 (95%CI = 0.986, 1.000), respectively. The accessibility to secondary care was positively associated with the odds of untreated hypertension for both slow and quick distance-decay weight: OR = 1.544 (95% CI = 1.303, 1.830), OR = 1.311 (95%CI = 1.163, 1.479). The interaction term of the accessibility of primary and secondary care facilities was negatively associated with the odds of untreated hypertension for both slow and quick distance-decay weight: OR = 0.970 (95%CI = 0.944, 0.996), OR = 0.975 (95%CI = 0.959, 0.991). In terms of demographic characteristics, females (vs. males) had lower odds, and smokers (vs. non-smokers) and drinkers (vs. non-drinkers) had higher odds of having hypertension from the advanced model with both slow and quick distance-decay weight: OR = 0.744 (95% CI = 0.703, 0.787), OR = 1.077 (95%CI = 1.021, 1.136), OR = 1.659 (95% CI = 1.581, 1.741). There was a positive association between individual BMI and the odds of having hypertension: OR = 1.188 (95%CI = 1.180, 1.195). There was a negative association between neighborhood income and the odds of having hypertension: OR = 0.999 (95%CI = 0.998, 0.999). For the result of untreated hypertension status, the direction of the association was the same as that of hypertension status in terms of gender but was the opposite for age and BMI. The odds of untreated hypertension decreased when age and BMI increased: OR = 0.928 (95% CI = 0.925, 0.932), OR = 0.922 (95%CI = 0.914, 0.931), respectively. There were no significant associations for the other covariates: smoking, drinking, and neighborhood income with untreated hypertension status.

**Table 3:** Multivariable logistic regression for hypertension and untreated hypertension status by primary and secondary care accessibility.

	Hypertension status				Untreated hypertension status			
	Slow distance-decay		Quick distance-decay		Slow distance-decay		Quick distance-decay	
	Model 1^a^	Model 2^a^	Model 1^b^	Model 2^b^	Model 1^a^	Model 2^a^	Model 1^b^	Model 2^b^
	OR (95% CI)		OR (95% CI)		OR (95% CI)		OR (95% CI)	
E2FCA score for primary care	**0.989**	1.003	**0.989**	0.999	0.994	1.01	0.993	1.007
	(0.984, 0.994)	(0.992, 1.013)	(0.984, 0.993)	(0.992, 1.007)	(0.987, 1.002)	(0.994, 1.026)	(0.986, 1.000)	(0.995, 1.018)
E2FCA score for secondary care	**1.281**	**1.758**	**1.130**	**1.469**	**1.544**	**2.226**	**1.311**	**1.839**
	(1.148, 1.430)	(1.392, 2.222)	(1.045, 1.221)	(1.250, 1.727)	(1.303, 1.830)	(1.547, 3.202)	(1.163, 1.479)	(1.430, 2.367)
E2FCA score for primary * secondary care		**0.974**		**0.981**	**0.970**			**0.975**
		(0.957, 0.991)		(0.970, 0.991)	(0.944, 0.996)			(0.959, 0.991)
Gender (ref = male)	**0.744**	**0.744**	**0.744**	**0.743**	0.911	0.910	0.910	0.910
	(0.703, 0.788)	(0.703, 0.787)	(0.702, 0.787)	(0.702, 0.787)	(0.828, 1.003)	(0.827, 1.002)	(0.827, 1.001)	(0.827, 1.002)
Age	**1.095**	**1.095**	**1.095**	**1.095**	**0.928**	**0.928**	**0.928**	**0.928**
	(1.092, 1.097)	(1.092, 1.097)	(1.092, 1.097)	(1.092, 1.097)	(0.925, 0.932)	(0.925, 0.932)	(0.925, 0.932)	(0.925, 0.932)
BMI	**1.188**	**1.188**	**1.188**	**1.188**	**0.922**	**0.922**	**0.922**	**0.922**
	(1.180, 1.195)	(1.180, 1.195)	(1.180, 1.195)	(1.180, 1.195)	(0.913, 0.931)	(0.913, 0.931)	(0.914, 0.931)	(0.914, 0.931)
Smoking (ref = no)	**1.078**	**1.077**	**1.078**	**1.077**	1.007	1.005	1.007	1.005
	(1.022, 1.137)	(1.021, 1.136)	(1.021, 1.137)	(1.021, 1.136)	(0.923, 1.098)	(0.922, 1.096)	(0.923, 1.098)	(0.922, 1.097)
Drinking (ref = no)	**1.660**	**1.659**	**1.660**	**1.659**	0.936	0.936	0.935	0.935
	(1.581, 1.742)	(1.581, 1.741)	(1.581, 1.742)	(1.581, 1.741)	(0.863, 1.015)	(0.863, 1.015)	(0.862, 1.014)	(0.862, 1.014)
Mean neighborhood income	**0.999**	**0.999**	**0.999**	**0.999**	1.000	1.000	0.999	1.000
	(0.998, 0.999)	(0.999, 0.999)	(0.998, 0.999)	(0.998, 0.999)	(0.999, 1.001)	(0.999, 1.001)	(0.999, 1.000)	(0.999, 1.000)
Observations	52,029	52,029	52,029	52,029	14,369	14,369	14,369	14,369
Log Likelihood	-24,448.36	-24,443.83	-24,452.96	-24,446.34	-8,981.68	-8,979.19	-8,984.55	-8,980.05
Akaike Inf. Crit.	48,914.73	48,907.65	48,923.93	48,912.68	17,981.36	17,978.38	17,987.10	17,980.09

Model 1: Multivariable logistic regression for dependent variables by E2FCA score for primary care, secondary care, and adjusted for covariates

Model 2: Multivariable logistic regression including interaction term of E2FCA score for primary and secondary care, and adjusted for covariates

a: E2FCA score with slow distance-decay weight

b: E2FCA score with quick distance-decay weight

Note: Bold shows significant p<0.05

## Discussions

In our study, we aimed to assess the association between hypertension status as well as treatment status and geographic accessibility to primary and secondary care facilities. The results indicate that geographic accessibility to primary care is important for workers in Shimane Prefecture in terms of both hypertension and treatment status. On the contrary, geographic accessibility to secondary care facilities was found to be associated with an increased risk of having hypertension and untreated hypertension. However, the model with the interaction term for primary and secondary care accessibility showed that the multiplicative effect of primary and secondary care accessibility decreased the risk of hypertension and untreated hypertension. The interaction could be interpreted as the effect of primary care accessibility was modified by secondary care accessibility, or vice versa. Theoretically, the effect of primary care accessibility may have mitigated the effect of secondary care accessibility as the hypertensive condition as well as treatment status are assumed to be affected by easy access to care but not expensive specialized care. Primary care facilities in Japan are usually open on weekends, while secondary care facilities generally are not and typically do not accept initial visits for hypertension except on weekday mornings. Therefore, when only secondary care is available close by, it may not increase the likelihood of workers seeking care unless they take extra time off, which rarely happens. However, greater access to primary care alone did not result in improved status for hypertension treatment. This may be because primary care service has not been fully established yet in Japan; thus, it might not be widely accepted and known of by the communities and residents. In addition, access to care is not determined by only geographic accessibility, but also aspects of care quality, such as continuity of care, physicians’ communication skills, and waiting time. These components could not be assessed in this study, and it could have overlooked some effect of primary care access on hypertension treatment status.

In terms of demographic characteristics, the analysis of hypertension status was consistent with principles related to access to care. The following components are known issues relevant to care access [1]: finances, geography, culture and society, education, gender, family, comorbidity, and health system type. The result of this study demonstrated how four of them—finances, geography, gender, and comorbidity—functioned as barriers to care access. Males, smokers, drinkers, and people with higher BMI had significantly higher odds of having hypertension than each of their respective counterparts. Those living in neighborhoods with lower incomes had higher odds of having hypertension. These findings indicate that healthcare provisions should focus on assisting people who have a higher risk of hypertension, financially as well as physically. These results are consistent with studies on health equity, which is one of the core principles of primary care as well as public health [20]. By contrast, the result for untreated hypertension status was not consistent with these common barriers. Age and BMI were negatively associated with the odds of untreated hypertension, and no associations were detected for smoking and drinking status. This finding could indicate that people who are young and fit may be reluctant to seek care for hypertension as it does not have immediate impacts on health. Such a finding is reflective of the Japanese health system, which offers universal health insurance and covers a population with a smaller socioeconomic gap than that in more multiracial countries without universal health insurance such as the United States. To our knowledge, this was the first finding to add a new perspective to principles of primary care considering overall population health improvements and a reduction in total health spending. Such a finding demonstrates how crucial it is that the government consider not only socially disadvantaged or high-risk groups but also healthy young adults along when deciding on and enacting ongoing policy changes concerning primary care in Japan. Region-specific findings are important because there are areas where health needs vary by type of disease and social and cultural backgrounds.

The major strength of this study was the assessment of geographic accessibility to healthcare using sophisticated spatial measures and individual health risk. By identifying the areas with health needs based on the actual association between geographic accessibility and individual health risk, medical resource allocation can be directed efficiently to achieve greater health equity. Another strength of this study was the assessment of the role of primary care, which, despite not being established, is moving toward distinct specialization of primary care establishment in Japan. The first official specialty training program of primary care started in April 2018, and the findings will be a useful guide to indicate the populations and areas that should be targeted.

There are several limitations to our study. First, the study only accounts for medication treatment when defining the untreated hypertension status. In order to effectively reduce the risk of hypertension complications, treatment should involve nonpharmacological therapy (also called lifestyle modification) as well as medication therapy [21, 22]. Second, while the E2FCA is a sophisticated form of spatial measure of healthcare accessibility, it still includes methodological limitations. Recent studies have been proposing a more enhanced model with multi-modal accessibility, or the model accounts for both spatial and non-spatial measures [23, 24]. As access to care entails non-spatial components such as waiting time and cost, these measures could potentially assess healthcare accessibility more precisely. Third, as primary care physicians are not established as a specialty in Japan, we used primary care facility as a potential provider of primary care. In the real world, there are doctors who function as primary care providers not only in primary care facilities but also in secondary care facilities, especially in areas where primary care facilities are not physically available. Lack of distinct data regarding primary care provision might have underestimated its effect in this study. Fourth, it is possible that other individual factors such as health literacy, education level, and family income might have had an impact on both hypertension and treatment status. We used neighborhood income level, smoking, drinking, and BMI as indicators of individual lifestyle and socioeconomic status; however, more detailed information would be desired for future studies. Fifth, we did not account for the presence of other serious comorbidities in the participants. It might have been possible that people with serious comorbidities were more likely to receive antihypertensive medications compared to those with only asymptomatic hypertension. Lack of control for these potential confounders might have led to overestimating of the healthcare accessibility effect on both hypertension and hypertension treatment status. Sixth, the participants were limited to workers, so the findings may not be generalizable to people who do not work. Finally, both hypertension status and untreated hypertension status were based on blood pressure measured once at the annual health checkup, and this study used a cross-sectional design. Further studies needed to identify causal relationships between hypertension and its treatment status and geographic healthcare accessibility using a longitudinal study design.

## Conclusions

In conclusion, geographic accessibility to primary care facilities was important for hypertension and untreated hypertension. In order to achieve health equity by establishing and maintaining equal accessibility to healthcare, it is essential to assess healthcare accessibility with appropriate spatial measures, consider its association with individual health risks, and gain region- and culture-specific perspectives.
